# Response of microbial biomass and CO_2_-C loss to wetting patterns are temperature dependent in a semi-arid soil

**DOI:** 10.1038/s41598-017-13094-9

**Published:** 2017-10-12

**Authors:** Yichao Rui, Deirdre B. Gleeson, Daniel V. Murphy, Frances C. Hoyle

**Affiliations:** 1 SoilsWest, UWA School of Agriculture and Environment, Faculty of Science, The University of Western Australia, Crawley, WA 6009 Australia; 2 Department of Agriculture and Food Western Australia, South Perth, WA 6151 Australia

## Abstract

One of the greatest contemporary challenges in terrestrial ecology is to determine the impact of climate change on the world’s ecosystems. Here we investigated how wetting patterns (frequency and intensity) and nutrient additions altered microbial biomass and CO_2_-C loss from a semi-arid soil. South-western Australia is predicted to experience declining annual rainfall but increased frequency of summer rainfall events when soil is fallow. Agricultural soils (0–10 cm at 10 °C or 25 °C) received the same total amount of water (15 mL over 30 days) applied at different frequency; with either nil or added nitrogen and phosphorus. Smaller more frequent wetting applications resulted in less CO_2_-C loss (*P* < 0.001); with cumulative CO_2_-C loss 35% lower than a single wetting event. This coincided with increased microbial biomass C at 25 °C but a decline at 10 °C. Increasing nutrient availability decreased CO_2_-C loss only under a single larger wetting event. While bacterial and fungal abundance remained unchanged, archaeal abundance and laccase-like copper monooxidase gene abundance increased with more frequent wetting at 25 °C. Our findings suggest smaller more frequent summer rainfall may decrease CO_2_ emissions compared to infrequent larger events; and enhance microbial C use efficiency where sufficient background soil organic matter and nutrients are available.

## Introduction

Soil is the largest terrestrial reservoir of carbon (C, 1500 Gt), containing twice as much as the atmosphere (780 Gt) and three times that in global vegetation (575 Gt)^1^. Microbially respired CO_2_ during soil organic matter (SOM) decomposition is an important source of atmospheric CO_2_ emissions, with small changes in soil respiration able to cause an ecosystem to switch from being a sink to a source of C. Therefore, alterations in soil respiration rate and soil C balance will have a significant impact on the global C cycle^2^.

Spatial and temporal patterns of water availability are the fundamental drivers of biological processes in arid and semi-arid ecosystems^3^. It is well documented that increased water availability can stimulate microbial activity and CO_2_-C loss from either microbial biomass or non-biomass due to a number of different mechanisms^4–6^. Rainfall events can cause soil aggregates to break apart, exposing previously physically protected SOM to decomposition^7^. Meanwhile, increases in water potential can not only cause microorganisms to rapidly oxidise cytoplasmic solutes, and release a large pulse of CO_2_^8^, but also induce osmotic shock, lyse extant microbial biomass, and release a pool of labile SOM that is used by surviving microorganisms^9^. Although the majority of ecosystem models assume a roughly linear response of SOM decomposition to increased water availability^10^, how shifts in rainfall patterns (timing, frequency, magnitude and duration) due to climate change impact on microbial populations and soil C dynamics remains unclear. Discrete rainfall events may trigger a series of soil responses at shorter and finer temporal and spatial scales, compared to rainfall patterns at broad time scales^11^. It has been suggested that increased rainfall variability under seasonally dry climates may decrease long-term C sequestration due to increased CO_2_ efflux induced by pulse-like events^8^. However, it is unknown how that same amount of seasonal rainfall applied at different frequency and intensity (smaller more frequent rainfall events, versus larger infrequent rainfall events) would affect soil respiration and microbial biomass and C use efficiency, and secondly whether under differing temperature this would either be negated or exacerbated by a rate change in microbial processes. Therefore, a better understanding of relationships between soil microbial activity and the size and timing of rainfall pulses at varied temperature is important for understanding how climate may influence the C balance of arid and semi-arid regions – particularly where wetter, warmer conditions are considered likely.

Microbial C dynamics and soil C storage are also influenced by nutrient availability which may regulate the response of microbial respiration to rainfall frequency and intensity^12^. The influence of adding inorganic nitrogen (N) on SOM decomposition rates can be positive, negative, or neutral^13^, with these effects largely dependent on the C:N ratio associated with the different stages of decomposition (i.e., early, late, and final) and thus changing requirements for additional nutrients. For instance, the application of ammonium (NH_4_^+^) and nitrate (NO_3_^−^) fertiliser to fresh litter stimulated the initial decomposition of cellulose and increased dissolved SOM, while the addition of the same compounds to humus clearly suppressed microbial activity and SOM decomposition^14^. Furthermore, nutrient addition to soil has been shown to slow microbial respiration and promote C storage over the long term^15–17^. A one-year incubation study with 28 soils from a broad range of ecosystems showed a consistent decrease in microbial respiration and biomass and a consistent shift of microbial community composition under N addition^17^.

Microorganisms under N enrichment may slow decomposition of recalcitrant C due to their lowered N requirements, with microorganisms no longer having to ‘mine’ recalcitrant SOM to obtain N^18^. Kirkby *et al*.^19^ also suggest that increased inorganic nutrient levels that meet the stoichiometric requirement of the microbial biomass for nutrient availability in regards to N, phosphorus (P) and sulphur (S) ratios would favour SOM stabilisation and thus could result in greater potential for C sequestration in soil. Given the potential influence of nutrient status on microbial C dynamics as well as C storage, understanding the interactive effects of wetting frequency and nutrient availability on microbial respiration will help predict the ecosystem functioning and C sequestration potential under future climate change scenarios.

Since the mid-1970s, the south-west of Western Australia (which is characterised by cool, wet winters and hot, dry summers) has experienced a 15% decrease in average annual rainfall^20^, and a further 5–11% decline is anticipated by 2050 compared to pre-1990 levels, with most decreases likely to be experienced during the winter months^20,21^. Climate predictions for increased temperatures and a continued shift to more frequent summer rainfall events would suggest a potential decline in soil organic C stocks for areas in this region which historically have already experienced a 15% decrease in heavy winter rainfall (1950–2003)^22^. Trends in rainfall characteristics are likely to be associated with changes in atmospheric circulation patterns, and may have a critical role in SOM decomposition^23^ and subsequently the C source/sink behaviour of the system^24^. These potential changes in rainfall distribution combined with alterations to nutrient availability are likely to influence microbially produced extracellular enzymes (for example cellulase and laccase that decompose cellulose and lignin respectively) that drive the decomposition of SOM^25^. However predicting these responses requires a better understanding of the role of rainfall timing and frequency across semi-arid landscapes.

**Figure 1:**
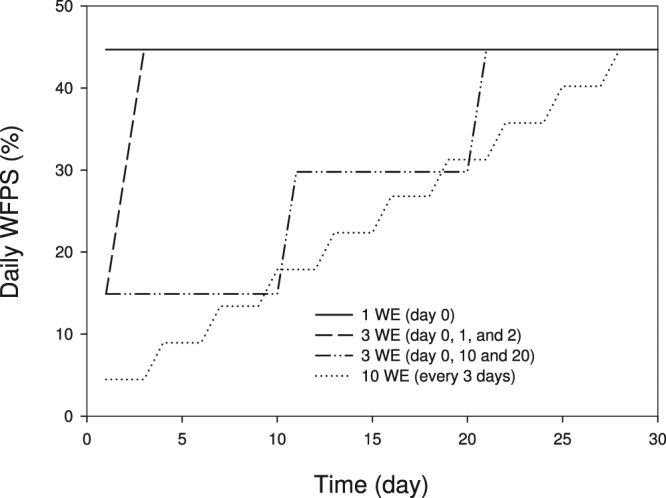
Theoretical soil water-filled pore space (WFPS) following wetting applications (WE) over the 30-day incubation period. Lines represent indicative soil water content on application of wetting treatments based on changes occurring within a day of application. Once treatments meet the upper water-filled pore space continuum (solid line) reflective of a single wetting event (1 WE) they remain steady to the end of incubation.

Here we investigated the responses of soil C dynamics, the total microbial biomass C (MBC), specific microbial populations, functional SOM decomposition gene abundance and CO_2_-C loss to variable rainfall frequency and intensity (Fig. 1) under either nutrient enriched (narrower C:N:P:S ratio) or constrained conditions within a semi-arid agricultural soil from Western Australia. Field soil was collected and incubated at 10 °C or 25 °C, to reflect average daily temperatures experienced for this region during the winter and summer months. Wetting treatments were imposed where soils received the same total amount of water (i.e., 15 mL over 30 days; equivalent to ~13 mm rainfall) applied at different times and frequency (Fig. 1); by two nutrient levels: ‘nil’ with no added N and P and ‘plus nutrients’ (plus N + P; 166 kg N ha^−1^, 40 kg P ha^−1^). In particular, we monitored the metabolic quotient *q*CO_2_ (CO_2_-C /MBC) and the microbial quotient (MBC/soil C) to explore microbial C use efficiency (CUE). We also measured the abundance of functional SOM decomposition genes (LMCO: laccase-like copper monooxidase (lignin oxidation), *cbhI*: fungal cellobiohydrolase (cellulose decomposition) and *GH48*: actinobacterial glycoside hydrolase (cellulose decomposition)) as they reflected the degradation of SOM compounds by functional microbial groups. We hypothesised that: (1) small, frequent wetting events would decrease total CO_2_-C loss compared to larger less frequent wetting events under nutrient enriched condition; (2) increased wetting frequency would favour microbial biomass under nutrient enriched condition; and (3) smaller more frequent wetting events would decrease SOM decomposition gene abundance therefore release less CO_2_-C.

## Results

**Figure 2:**
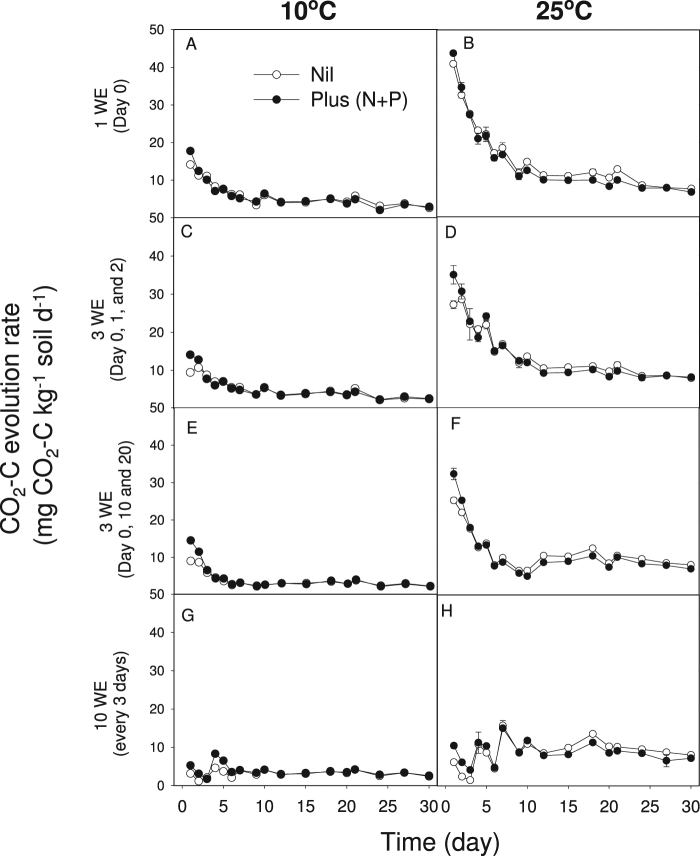
CO_2_-C evolution rate in soils received different wetting applications (WE) including one wetting on day 0 (**A** and **B**), 3 wettings on day 0, 1, or 2 (**C** and **D**), 3 wettings on day 0, 10, or 20 (**E** and **F**), 10 wettings on day 0, 3, 6, 9, 12, 15, 18, 21, 24, and 27 (**G** and **H**), and nutrients applications incubated at 10 °C and 25 °C during the 30-day incubation period. Bars represent standard errors.

Temperature had a strong influence on soil respiration with consistently (*P* < 0.001) higher daily CO_2_-C evolved at 25 °C than at 10 °C (Fig. 2). This resulted in higher total cumulative CO_2_-C after 30 days at 25 °C (P < 0.001, 317 ± 2 mg CO_2_-C kg^−1^ soil) compared to at 10 °C (115 ± 2 mg CO_2_-C kg^−1^ soil), and an average temperature coefficient for soil (Q_10_) of approximately 1.8 (range 1.7–2.0). This temperature influence dominated the interaction observed with the nutrient application for cumulative CO_2_-C (*P* < 0.001, Table 1).

**Table 1:** Properties including total carbon (C), total nitrogen (N), C:N ratio, and inorganic phosphorus (P), potassium (K), and sulphur (S) concentration of soils received different wetting applications and nutrients applications after 30 days of incubation at 10 °C and 25 °C (standard errors are shown in parentheses, n = 4; *indicates a significant difference at *P* < 0.05).

Temperature	Nutrients	Wetting # applications	Cumulative CO_2_-C evolution (mg CO_2_-C kg^−1^ soil)	Total C (%)	Total N (%)	C:N Ratio	Inorganic P (mg kg^−1^)	Inorganic K (mg kg^−1^)	Inorganic S (mg kg^−1^)
10 °C	Nil	1 (day 0)	143 (1)	0.86 (0.02)	0.07 (0.001)	12.2 (0.1)	24 (1)	103 (4)	6.2 (0.2)
		3 (day 0, 1, and 2)	122 (2)	0.85 (0.02)	0.07 (0.003)	11.7 (0.4)	29 (1)	119 (3)	6.7 (0.2)
		3 (day 0, 10, and 20)	97 (1)	0.86 (0.03)	0.08 (0.006)	11.1 (0.7)	29 (1)	130 (6)	6.5 (0.3)
		10 (every 3 days)	86 (1)*	0.88 (0.01)	0.08 (0.003)	11.1 (0.3)	31 (1)*	122 (4)	6.7 (0.3)
	Plus (N + P)	1 (day 0)	142 (2)	0.86 (0.03)	0.08 (0.003)	11.1 (0.4)	32 (3)	114 (6)	6.5 (0.4)
		3 (day 0, 1, and 2)	128 (2)	0.88 (0.03)	0.09 (0.005)	10.2 (0.4)	36 (0)	131 (7)	7.3 (0.3)
		3 (day 0, 10, and 20)	104 (1)	0.90 (0.04)	0.08 (0.003)	10.9 (0.3)	36 (4)	131 (5)	7.4 (0.6)
		10 (every 3 days)	100 (1)*	0.95 (0.02)*	0.09 (0.003)*	10.8 (0.3)	37 (1)*	130 (4)	6.6 (0.1)
25 °C	Nil	1 (day 0)	382 (8)	0.82 (0.01)	0.08 (0.002)	10.4 (0.2)	34 (1)	126 (3)	9.1 (0.4)
		3 (day 0, 1, and 2)	355 (8)	0.87 (0.04)	0.08 (0.003)	11.2 (0.4)	33 (2)	121 (5)	8.7 (0.1)
		3 (day 0, 10, and 20)	303 (2)	0.89 (0.02)	0.08 (0.001)	10.8 (0.3)	33 (2)	122 (7)	8.4 (0.6)
		10 (every 3 days)	260 (4)*	0.90 (0.03)	0.08 (0.004)	11.1 (0.2)	34 (1)	124 (4)	8.3 (0.3)
	Plus (N + P)	1 (day 0)	356 (4)	0.86 (0.02)	0.08 (0.004)	10.5 (0.5)	32 (2)	112 (3)	7.3 (0.4)
		3 (day 0, 1, and 2)	349 (4)	0.85 (0.03)	0.08 (0.003)	10.5 (0.2)	42 (2)	135 (2)	8.4 (0.4)
		3 (day 0, 10, and 20)	286 (2)	0.89 (0.03)	0.08 (0.003)	10.6 (0.2)	38 (1)	130 (4)	9.2 (0.3)
		10 (every 3 days)	245 (4)*	0.91 (0.03)	0.09 (0.003)	10.6 (0.1)	35 (2)*	117 (8)*	8.9 (0.4)

The influence of nutrients on daily CO_2_-C production rate was dependent on time of measurement and wetting treatment, with increased CO_2_-C evolved from nutrient enriched treatments (*P* < 0.001) during the first two days of incubation regardless of the wetting application (Fig. 2). In nutrient enriched treatments incubated at 25 °C,﻿ CO_2_-C evolved ﻿declined after seven days (Fig. 2). A significant influence (*P* < 0.001) of nutrients on cumulative CO_2_-C was only evident for soils receiving a single wetting application of 15 mL, with 5% less CO_2_-C on average being respired after application of N + P (249 mg CO_2_-C kg^−1^ soil) compared to nil soils (263 mg CO_2_-C kg^−1^, *P* = 0.04, LSD = 8).

The highest amount of cumulative CO_2_-C evolved was for a single wetting application with no added nutrients incubated at 25 °C (Table 1). Cumulative CO_2_-C declined (*P* < 0.001) with a lesser intensity of wetting (meaning either decreasing volumes of water at each application or longer intervals between wetting events; Table 1). Smaller more frequent wetting events (10 applications of 1.5 mL) evolved 32% less cumulative CO_2_-C after 30 days on average than a single application of 15 mL (Table 1). Increasing the interval between wetting treatments (3 applications of 5 mL) from one day to 10 days also decreased cumulative CO_2_-C by 17% on average (Table 1).

Interactions between the main treatment effects of wetting (*P* < 0.001), nutrient (*P* = 0.011) and temperature (*P* < 0.001) were evident for the amount of cumulative CO_2_-C evolved (Table S1) and reflected differences in daily CO_2_-C production rate measured on a continuous basis (Fig. 2).

With the exception of those treatments receiving 10 wetting applications of 1.5 mL water which were unchanged from initial total C concentration in soil (0.94%), a significant loss (*P* = 0.003, LSD = 0.04) in total C was associated with remaining wetting treatments (0.85–0.88%). No significant effect (*P* = 0.05) of temperature or nutrients on total C was noted. Total N was nominally higher (*P* < 0.001) in nutrient enriched treatments (0.083 ± 0.002%) compared to no added nutrients (0.079 ± 0.002%). This was accompanied by a resultant decline in the C to N ratio from 11.2 to 10.7 (*P* = 0.002), which was the same change observed for an increase in incubation temperature.

**Figure 3:**
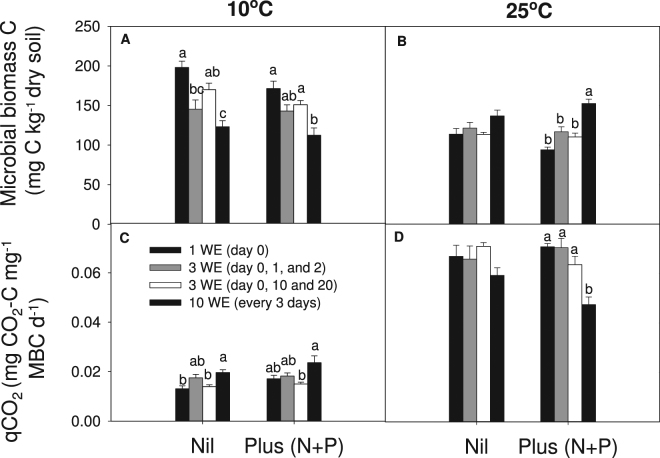
Microbial biomass C (MBC; (**A** and **B**) and metabolic quotient (*qCO*_*2*_, (**C** and **D**) of soils received different wetting applications (WE) and nutrients applications after 30 days of incubation at 10 °C and 25 °C. Bars represent standard errors. Different letters mean significant difference (*P* < 0.05) between wetting applications of this temperature × nutrient treatment.

After 30 days’ incubation, MBC was significantly higher at 10 °C (152 mg  C  kg^−1^ dry soil) than 25 °C (120 mg C kg^−1^ dry soil) (*P* < 0.001; Fig. 3). A significant interaction between wetting treatment and temperature (*P* < 0.001), showed MBC generally decreased with more frequent wetting applications at smaller volumes when incubated at 10 °C, but had the opposite effect at 25 °C (Fig. 3). This resulted in 39% higher MBC at 25 °C with 10 applications of 1.5 mL (145 mg C kg^−1^ dry soil), compared to a single 15 mL wetting event (104 mg C kg^−1^ dry soil). By comparison, MBC was lower in nutrient enriched treatments (132 mg C kg^−1^ dry soil) compared to no added nutrients (140 mg C kg^−1^ dry soil, LSD = 7) and showed no interaction with either wetting treatment or temperature.

The metabolic quotient *q*CO_2_ was higher at 25 °C than 10 °C (*P* < 0.001). Whereas, *q*CO_2_ increased with increasing wetting frequency at 10 °C, but decreased with the wetting frequency at 25 °C. At 25 °C, the metabolic quotient was 12% lower under 10 applications of wetting than under one application of wetting when no nutrient was added (*P* > 0.05), and 33% lower under 10 applications of wetting than under one application of wetting when nutrient was added (*P* < 0.001; Fig. 3).

**Figure 4:**
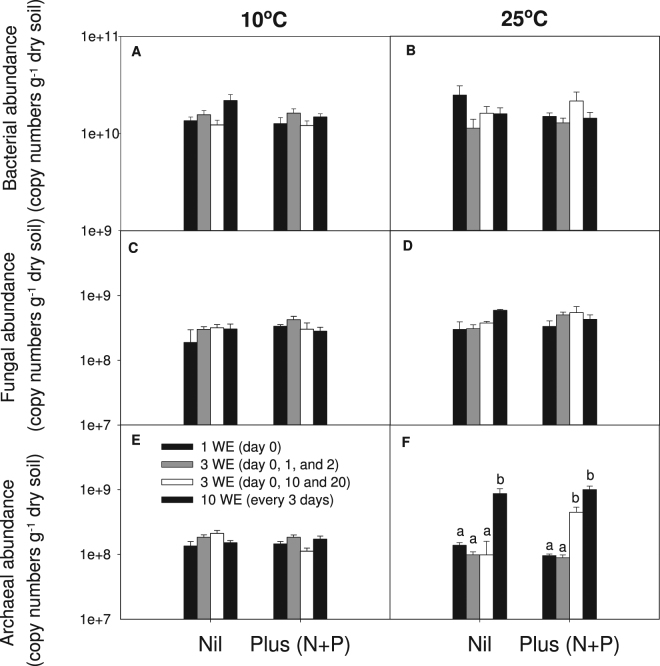
Abundance of total bacteria (**A** and **B**), fungi (**C** and **D**), and archaea (**E** and **F**) of soils that received different wetting applications (WE) and nutrients applications after 30 days of incubation at 10 °C and 25 °C. Bars represent standard errors. Different letters mean significant difference (*P* < 0.05) between wetting applications of this temperature × nutrient treatment.

After 30 days’ incubation there was no significant effect of temperature (*P* > 0.05), wetting frequency (*P* > 0.05) or nutrient amendment (*P* > 0.05) on bacterial and fungal abundance which averaged 1.6 × 10^10^ and 3.6 × 10^8^ gene copies g^−1^ dry soil, respectively (Fig. 4). While there was no overall significant effect of wetting frequency (*P* > 0.05), temperature (*P* > 0.05) or nutrient amendment (*P* > 0.05) on archaeal abundance, there was a significant interaction (*P* < 0.05). At 25°C, archaeal abundance was significantly higher with increased wetting frequency (*P* < 0.01) while at 10°C there was no significant effect of wetting frequency (*P* > 0.05). Regardless of the nutrient amendment, archaeal abundance was significantly higher (*P* < 0.01) under small frequent wetting application (every 3 days throughout the incubation) than low wetting frequency (one wetting event at day 0 and 3 wetting events either at day 0, 1 and 2 or 0, 10 and 20).

**Figure 5:**
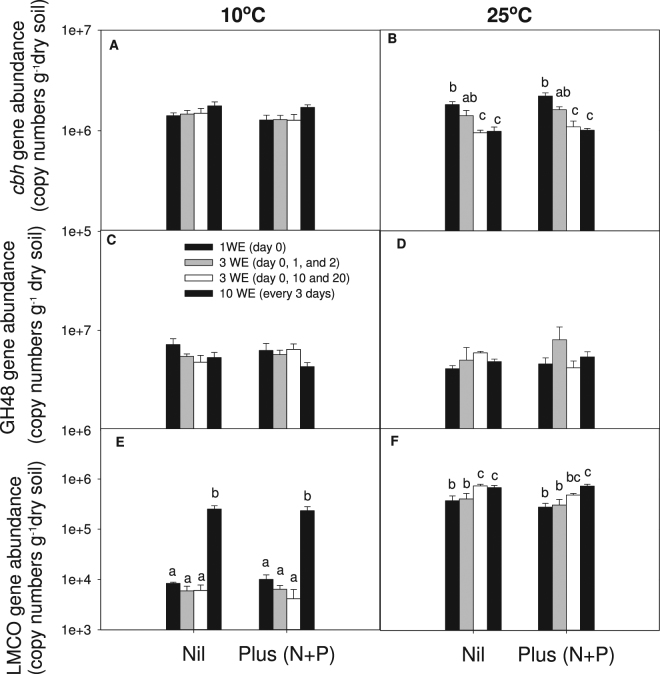
Abundance of functional genes *cbhI* (**A** and **B**), *GH48* (**C** and **D**) and LMCO (**E** and **F**) of soils received different wetting applications (WE) and nutrients applications after 30 days of incubation at 10 °C and 25 °C. Bars represent standard errors. Different letters mean significant difference (*P* < 0.05) between wetting applications of this temperature × nutrient treatment.

At 10 °C there was no significant effect of wetting frequency (*P* > 0.05) on the abundance of *cbhI*, fungal cellobiohydrolase (Fig. 5). At 25°C *cbhI* gene abundance was significantly (*P* < 0.01) lower with 10 wetting applications (1.0 × 10^6^ copies g^−1^ dry soil) compared to one wetting application (2.0 × 10^6^ copies g^−1^ dry soil). There was no significant effect of nutrient amendment *(P* > 0.05) on the abundance of *cbhI* despite the significant interaction between temperature and wetting frequency (*P* < 0.05). There was no significant effect of temperature (*P* > 0.05), wetting frequency (*P* > 0.05) or nutrient amendment (*P* > 0.05) on the abundance of *GH48*, Actinobacterial glycoside hydrolase, which averaged 5.5 × 10^6^ copies g^−1^ dry soil. The abundance of LMCO, fungal and bacterial laccase-like copper monooxidase, was significantly higher (*P* < 0.05) under more frequent wetting applications at both 10°C and 25°C. At 10°C there was a significant increase in LMCO gene abundance from 9.2 × 10^3^ copies g^−1^ dry soil under one wetting application to 2.4 × 10^5^ copies g^−1^ dry soil under 10 wetting applications. LMCO gene abundance at 25°C was significantly higher than at 10°C and significantly higher (*P* < 0.05) under more frequent wetting applications. There was no significant effect of the nutrient amendment (*P* > 0.05) on the abundance of LMCO, despite a significant interaction between temperature and wetting frequency (*P* < 0.05).

Ammonium-N concentration was less than 1 mg N kg^−1^ (data not shown) across all treatments and NO_3_^-^-N was the major form of inorganic N present. Nitrate-N levels were higher (*P* < 0.001) at 25 °C (51 mg N kg^−1^ dry soil) than 10 °C (32 mg N kg^−1^ dry soil); and in nutrient enriched treatments (60 mg N kg^−1^ dry soil) compared to nil treatments (23 mg N kg^−1^ dry soil).

**Figure 6:**
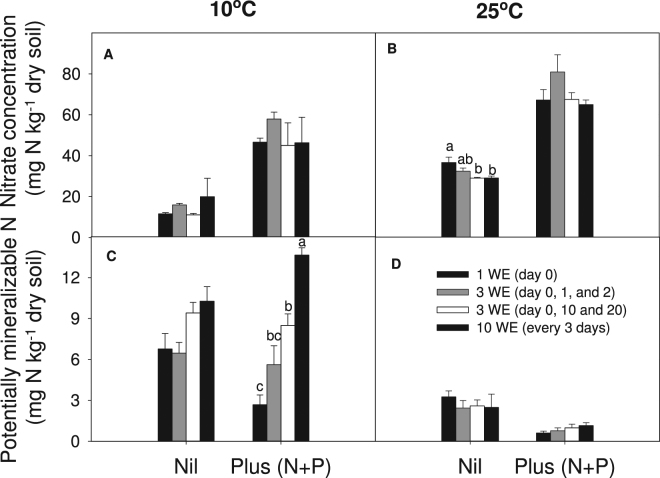
Nitrate-N concentration (**A** and **B**) and potentially mineralisable N (**C** and **D**) of soils received different wetting applications (WE) and nutrients applications after 30 days of incubation at 10 °C and 25 °C. Bars represent standard errors. Different letters mean significant difference (*P* < 0.05) between wetting applications of this temperature × nutrient treatment.

A significant interaction (*P* = 0.047) between temperature, nutrients and wetting treatments was observed for potentially mineralisable N (PMN) (Fig. 6). Potentially mineralisable N was higher for all treatments when incubated at 10 °C (average 7.9 mg N kg^−1^ dry soil) than at 25 °C (average 1.8 mg N kg^−1^ dry soil), and increased with smaller, more frequent wetting applications only when incubated at 10 °C (Fig. 6). When incubated at 25 °C, PMN was relatively low and there was little change evident with different wetting treatments (Fig. 6).

A significant interaction between temperature and nutrient treatments resulted in a small increase (*P* = 0.05) in P levels being measured at 10 °C where soil had no nutrients added, but no additional influence of temperature on P where soils had been nutrient enriched (Table 1). Higher P in treatments which had been nutrient enriched was only evident for soils incubated at 10 °C (*P* = 0.05). Multiple smaller wetting events resulted in greater P remaining at the end of the incubation than those receiving a single wetting event (*P* = 0.003).

A significant interaction between temperature, wetting, and nutrients was observed for inorganic S (*P* < 0.05). In general, higher levels (*P* = 0.045) of S were observed in soils after incubation at 25 °C than at 10 °C (Table 1, Table S1) with only one exception across all treatments. In soils where no nutrients were added and those incubated at 10 °C, S levels remained unchanged regardless of wetting treatment. At 25 °C, soil S increased nominally in other treatments compared to a single wetting event (Table 1, Table S1). Potassium (K) concentrations were high in this soil (average 123 mg kg^−1^ dry soil) and further increased (*P* < 0.001) in treatments with more than a single wetting event.

## Discussion

Although all treatments had the same total amount of water applied by the end of incubation, our results show a variable response of CO_2_-C efflux to wetting size and frequency. Temperature had the strongest influence on evolution of CO_2_-C across all treatments with an average Q_10_ of 1.8, demonstrating higher rates of SOM decomposition under warmer temperatures. Smaller more frequent wetting applications resulted in less cumulative CO_2_-C loss and at higher temperatures greater microbial biomass compared to larger or less frequent wetting applications. This suggests higher microbial C use efficiency and consequently greater soil C sequestration potential of this soil under smaller more frequent rainfall scenarios, which we suggest is associated with a smaller volume of soil being influenced by each wetting event. It has been previously suggested that different amounts of rainfall input to a dry soil can trigger various magnitudes of response for biogeochemical transformations. Part of this response is likely dependent on the extent (depth and location) of soil directly affected by initial and subsequent wetting events. Small pulses of wetting events may only affect a small volume of soil and trigger relatively minor ecological events, while larger wetting pulses may affect a larger volume of soil and trigger larger ecological events^11^. It is possible that many large biological state changes such as depolymerisation and lysis of microbial biomass, have a minimum requirement to trigger such events^11^. The greater cumulative CO_2_-C loss under larger less frequent wetting events in this study may be a direct result of the large pulse of CO_2_ at the beginning of the incubation as the wetting size was big enough to trigger many biological processes and influence a greater volume of soil. A rapid pulse of CO_2_ efflux following rainfall and rewetting of dry soil has been observed in a variety of ecosystems^8,26,27^. However compared to a large magnitude rainfall event, microbial activity following smaller rainfall events are likely to be short-lived and subsequent C losses relatively small^11^. Our study was different from dry-wet cycle studies in which soils experienced repeatedly shifting water potential and osmotic shock, and usually with enhanced aggregate turnover^28^. Instead, our study demonstrated that gradual increase of soil moisture under smaller more frequent wetting events, could result in less CO_2_ efflux, compared with large infrequent wetting pulses.

An increase in microbial biomass to rainfall events is a well-documented response in arid ecosystems^29,30^. Our results suggested smaller more frequent rainfall events favour microbial biomass compared to larger infrequent rainfall events at 25 °C. Sudden intense changes in moisture associated with infrequent rainfall are stressful to microorganisms, as they must expend energy to regulate osmotic pressure to their microenvironment, while smaller but more frequent wetting creates an increase in conditions that are conducive to biological activity^31,32^. Parker *et al*.^33^ observed that MBC peaked six days after a simulated rainfall event and then started to decline in a desert soil. However, under smaller more frequent wetting events at 25 °C in this experiment, the labile substrate was either consumed more slowly than in larger wetting events thereby maintaining microbial growth under smaller, more frequent water pulses; or influenced a smaller proportion of the soil volume and thus had a lesser impact on the microbial biomass. By contrast, at 10 °C when microbial metabolic activity was lower, MBC was higher under larger infrequent wetting events. This may imply that under low temperature labile SOM substrate was not depleted as quickly and thus the microbial activity was mainly controlled by water availability.

After 30 days there were no significant treatment effects on the abundance of bacteria or fungi, and only minimal effects on archaeal abundance (increased wetting frequency at 25°C significantly increased archaeal abundance). This is not surprising as the effects of wetting and nutrients are more likely to be mediated through changes in community structure than overall abundances^34^. Additionally it is well established that microbial communities are likely the fastest components of an ecosystem to respond to changing conditions^35,36^ and thus in assessing these populations after 30 days it is likely that any changes in abundances that had occurred would have taken place immediately after treatments were applied and were no longer observable on completion of the experiment. The unchanged bacterial and fungal abundance, but greater MBC under smaller more frequent wetting treatments at 25 °C shows microbial populations could have incorporated more C in forming microbial biomass under smaller more frequent wetting and nutrient enriched conditions. Taken together, the stimulated microbial growth by smaller frequent water pulses at 25 °C implies greater C stabilisation potential through the formation of organo-mineral association because stable SOM is formed predominantly from microbial detritus and the microbial pathway of organo-mineral formation has been increasingly recognised as the principal mechanism for long-term C accumulation^37^. Although the impact of wetting frequency on long-term C storage needs more investigation by conducting large-scale experiments, the small but significant decreased loss of soil total C (*P* = 0.015) under increased frequency at both incubation temperatures implies the potential to sequester C in agricultural soils in the southwest of Western Australia under future climate conditions (increasing frequency in summer-autumn rainfall).

Smaller more frequent wetting events led to higher inorganic P and K concentrations than a single wetting event at 10 °C. This could be attributed to enhanced decomposition of the lignin component of SOM at 10 °C, as reflected by higher LMCO gene abundance (lignin oxidation) under smaller more frequent wetting treatments; decomposition of the cellulose component of SOM was unaffected by wetting frequency. This increase in LMCO abundance may also reflect the greater capacity of the community to decompose more recalcitrant SOM (e.g. lignin component) under increased wetting frequency which has further consequences for our ability to sequester C where changing climate scenarios predict an increased frequency of rainfall events. Meanwhile, given the decrease of microbial biomass under smaller more frequent wetting events at 10 °C, the inorganic P release could also be directly from microbial biomass^38^ on wetting events. As soil water potential increases after rainfall events, microorganisms must release solutes before increased osmotic pressure leads to bursts cells^39^. This suggests water availability regulated microbial biomass can act as a short-term reservoir of available nutrients. As a result, smaller more frequent wetting could lead to greater inorganic nutrient supply.

The magnitude of the effect of wetting applications on soil C sequestration may depend on the nutrient availability. Nutrient inputs suppressed cumulative CO_2_-C loss in the present study while the daily CO_2_-C production showed an increase under nutrient addition at the beginning of incubation but a decrease after 5 days of incubation. This corresponds with Berg and Egbert^14^ who have suggested that the effects nutrient addition have on microbial activity tend to differ depending on the stage of decomposition. However, Ramirez *et al*.^17^ suggest that in the long run, nutrient addition tends to suppress the microbial respiration by similar wide-spread mechanisms regardless of climate and soil characteristics. For example, N additions could directly inhibit enzymes needed for the decomposition of recalcitrant C thereby decreasing overall microbial activity^40^. However, in the present study we did not observe significant effects of nutrient addition on gene abundance of any SOM genes assessed (cellulose or lignin-related) which is in line with Keeler *et al*.^41^ who reported neutral effects of nutrient addition on SOM decomposition associated enzyme activities. As a result, the nutrient addition induced shift in microbial respiration in this study might be attributed to the lowered N requirements of microorganisms^18^. This shift might occur at the level of the individual microbe and/or be a consequence of shifts in the relative abundances of specific microbial taxa. It has been reported that nutrient additions could decrease microbial activities by directly shifting microbial community composition^42–44^, as microbial groups that have fast growth rates and rely on more labile C sources are more likely to increase in abundance under nutrient inputs, while other groups that likely thrive under lower nutrient conditions and grow more slowly would decline^42^. As a consequence, the N-induced shifts in microbial community structure should yield corresponding shifts in the functional and metabolic potential of the communities, and in this case, resulting in a change in decomposition rates. Our results suggested that, under large infrequent rainfall conditions, nutrient input could decrease the CO_2_-C loss as a result of the decreased microbial respiration.

Different to Ramirez *et al*.^17^ who observed a consistent decrease in microbial biomass under nutrient addition, we found under smaller more frequent wetting (10 applications) at 25 °C, MBC increased with nutrient addition. Effects of wetting frequency on MBC were amplified by nutrient inputs. Likewise, lower *q*CO_2_ in response to nutrient addition under smaller more frequent rainfall (10 applications) at 25 °C demonstrate the higher efficiency of microorganisms in C utilisation. The interaction between wetting applications and nutrient input has implications for understanding the soil microbial C dynamics under climate change scenarios, and show the combination of nutrient input and increasing wetting frequency would favour microbial growth in soil. This may lead to greater C sequestration as the microbial pathway of organo-minerals formation has been increasingly recognised as a driving mechanism for long-term C accumulation. Combined these suggest that smaller more frequent summer rainfall events may decrease the potential for greenhouse gas emissions compared to infrequent larger events, and enhance microbial utilisation efficiency of C in soils where sufficient background soil organic matter is available.

## Conclusion

We show that smaller more frequent summer rainfall events may decrease CO_2_-C loss at both 10 and 25°C. Enhanced microbial C use efficiency in soils was also noted at 10 °C associated with slower decomposition compared to 25 °C. Changes in MBC to increased wetting frequency were temperature dependent and decreased under enriched nutrient conditions. While results reinforce the complexity of responses resulting from the changing climate, they suggest that smaller but more frequent summer rainfall events (as predicted to occur in south-west Western Australia) will slow C loss from soils in the absence of growing plants. These findings support our initial hypotheses on the influence of wetting frequency which appears relatively ubiquitous, but is in contrast to the response to nutrient enriched conditions which only had a minor influence when sufficient moisture was present. Our results have implications for understanding soil C dynamics and C sequestration potential in future climate change scenarios in semi-arid and arid environments of south-west Western Australia that are experiencing climate change patterns.

## Materials and Methods

### Field trial site and soil collection

The soil was a free-draining sand classified as a Basic Regolithic Yellow-Orthic Tenosol (Isbell, 2002) or Haplic Arenosol^45^ that was collected from an experimental site (10 years) at Buntine (30.01 S, 116.34 E; elevation of 315 m) in south-west Western Australia. The region has a semi-arid climate with hot, dry summers and cool, wet winters (when cropping occurs). The nearby town of Dalwallinu had a mean annual rainfall of 290 mm, mean monthly minimum and maximum temperature of 5.8 °C to 35.5 °C respectively, with actual daily temperatures ranging from −1.0 to 46.9 °C (calculated from 2003 to 2014; http://www.bom.gov.au/climate/data).

Soil was collected from a continuously cropped, tilled field experiment treatment established in 2003. Prior to sampling the recent land-use history of the site was: 2010, wheat (*Triticum aestivum*); 2011, wheat (*T. aestivum*); 2012, canola (*Brassica napus*); 2013, barley (*Hordeum vulgare*); 2014, oats (*Avena sativa*). The soils we used in the incubation had a history of external organic matter input (80 t ha^−1^ added as chopped plant residues [chaff] over 10 years from 2003) and therefore had a wide C:N:P:S ratio. The initial ammonium N, nitrate N, inorganic P, inorganic S of field soil were 3, 21, 40 and 21 mg kg^−1^, respectively. Soil (0–10 cm) was collected manually from the Ap horizon in a zig-zag sampling pattern using a push-in sand auger in late October 2014; 120 cores were combined to make a single composite sample (7 cm diameter by 10 cm depth, 40 cores from each of three field replicates). Soils were dried at 40 °C for seven days prior to being sieved (4 mm sieve) and mixed thoroughly for a subsequent incubation study. Basic soil properties (0–10 cm) were 0.94% total C, soil pH 6.2 in CaCl_2_ and 6% clay.

### Sample preparation and incubation

Soil samples (100 g dry weight equivalent) were weighed into air-tight, 500 mL glass jars and sealed using lids modified with gas septum ports for seven days pre-incubation at 10 °C or 25 °C. Sufficient jars were pre-incubated for application of nutrient by wetting treatments in a complete factorial design (temperature × nutrient × wetting) with four replicates. Following pre-incubation two nutrient treatments (nil, plus N + P) were applied to represent nutrient constrained (nil, 0 N, 0 P) and enriched conditions (plus N + P; 166 kg ha^−1^ N, 40 kg ha^−1^ P). Nutrients added were applied dry to the soil as Urea (0.025 g per 100 g^−1^ dry soil per jar) and Triple Superphosphate (TSP; 0.014 g per 100 g^−1^ dry soil per jar) and mixed. Due to the exceptionally high rates of nutrients needed to meet required stoichiometric ratio (N, P, S) for complete C sequestration^19^, the enriched nutrient treatment was designed to meet the stoichiometric requirements for soil available S, and was equivalent to just 15% of the fertiliser required for complete C sequestration. Four wetting treatments were subsequently applied by pipette in single droplets to each of the jars over a 30-day incubation. These included a single application of 15 mL water (~13 mm equivalent rainfall, 45% water holding capacity) reflecting the average monthly rainfall received during November through to March (2003–2011) for the field site and applied on the first day (day 0). Other wetting treatments included 5 mL water applied on each of the first 3 days (day 0, 1, and 2); 5 mL water applied every 10 days (3 applications on day 0, 10, and 20); and 1.5 mL water applied every 3 days (10 applications on day 0, 3, 6, 9, 12, 15, 18, 21, 24, and 27) so that all treatments received the same amount of water (Fig. 1). A 5 mL vial of water was placed into the jars to minimise any potential soil drying and treatments were then incubated at either 10 °C or 25 °C (to approximate the average minimum and maximum temperatures (2003–2013) during the growing season (April-October) experienced in this region. Each treatment was replicated four times, and all soil samples were incubated for 30 days. Figure 1 shows a conceptual diagram of soil water-filled pore space (WFPS) for wetting treatments over the 30-day incubation period.

### CO_2_-C evolution

CO_2_-C evolved from the soil was measured in the headspace of the jars twice a week using an infrared gas analyser (IRGA). Headspace gas samples (1 mL of air from each jar, collected after first mixing the headspace) were analysed against a CO_2_ standard (4.95 ± 0.10% CO_2_ in helium, BOC Ltd.). The CO_2_-C results presented are the average daily rate of evolution measured for each treatment, minus a control treatment (no soil) to account for the CO_2_ concentration already in the jar headspace. After each sampling, all jars were opened and the headspace gas exchanged with fresh air.

### Soil analyses

After 30 days of incubation, soil from each of the jars was analysed for a range of soil properties. Soil total C and N were determined on air dried, finely ground soil by total combustion using a C/N-analyser (Elementar Vario Macro CNS, Hanau, Germany). Soil NH_4_^+^-N and NO_3_^-^-N concentrations were determined using 20 g of fresh soil shaken in 80 mL of 0.5 *M* K_2_SO_4_ for 1 h and filtered (Whatman #42). Ammonium-N and NO_3_^-^-N concentrations in the K_2_SO_4_ extracts were analysed colorimetrically by automated segmented flow auto-analysis (OI Analytical, College Station, Texas USA). Microbial biomass C was determined by fumigation extraction using 20 g (oven-dried equivalent) of soil shaken in 80 mL of 0.5 *M* K_2_SO_4_ for 1 h. Non-fumigated soils were also extracted. Oxidisable C in the K_2_SO_4_ extracts was determined using an Aurora 1030 Total Oxidisable C analyser (OI Analytical, College Station, Texas, USA). A *k*_EC_ factor of 0.45 was used to calculate the MBC. The metabolic quotient *q*CO_2_ (CO_2_-C /MBC) has been calculated to study microbial C use efficiency^46^.

Potentially mineralisable N was determined by anaerobic incubation^47^. Fresh soil (20 g) was incubated in 80 mL of Milli-Q water for 7 d at 40 °C. Potassium sulphate was added to adjust the soil solution to 0.5 *M* K_2_SO_4_, then samples were shaken for 1 h. Samples were analysed for NH_4_^+^-N after filtering as described above.

Inorganic P and K were measured using the Colwell method^48^. Soils were extracted with 0.5 *M* sodium bicarbonate solution adjusted to pH 8.5 for 16 hours. The acidified extract was treated with ammonium molybdate/antimony trichloride reagent and the inorganic P measured colorimetrically at 880 nm using a discrete analyser. The inorganic K in the extract was determined using a flame atomic absorption spectrophotometer at 766.5 nm. Inorganic S in soils was determined by extraction with a 0.25 *M* potassium chloride solution for 3 hours at 40 °C. The S content of extracts were analysed by inductively coupled plasma spectrometry (ICP). Soil pH was determined on air-dried soil (10 g) in 50 mL of CaCl_2_ (0.01 *M*), shaken for 1 h and left to stand overnight.

### DNA extraction and qPCR

After 30 days of incubation duplicate DNA extractions were performed on all soils using the Power Soil DNA isolation kit (MoBio Laboratories Inc.) following the manufacturer’s instructions. DNA from pooled extractions was quantified using a Qubit™ (Thermo Fisher Scientific) and diluted with sterile water to a concentration of 5 ng µL^−^^1^ for all subsequent quantitative polymerase chain reaction (qPCR) assays.

The abundance of genes encoding *16 S rRNA* gene (total bacterial and archaeal numbers), *18 S rRNA* (total fungal numbers) and functional SOM decomposition genes (LMCO, *cbhI*, and *GH48*) were determined by qPCR using an Applied Biosystems ViiA7 machine (Thermo Fisher Scientific); all samples were run in triplicate. Specific primers and qPCR cycling conditions are listed in Table S2. For bacterial and archaeal *16 S rRNA* and fungal *18 S rRNA genes*, each 20 µL qPCR reaction contained 10 µL of Power SYBR Green Master Mix (Applied Biosystems, Thermo Fisher Scientific), 0.2 µL of the specific forward and reverse primers at a concentration of 10 µ*M*, 2 µL bovine serum albumin (Ambion Ultrapure BSA, 5 mg mL^−1^), 2 µL of template DNA and 5.6 µL sterile water. The qPCR reaction for both LMCO and *GH48* was the same as described above except that Sso Q-PCR SYBR (Biorad) was used. For *cbhI*, each 20 µL qPCR reaction contained 10 µL of SensiFAST SYBR Lo-ROX (Bioline), 2 µL of the specific forward and reverse primers at a concentration of 10 µ*M*, 2 µL bovine serum albumin (Ambion Ultrapure BSA, 5 mg mL^−1^), 2 µL of template DNA and 2 µL water.

Templates for determining gene copy numbers in the qPCR reactions were cloned plasmids. PCR amplicons of all genes were cloned using P-GEMT (Promega, Madison, WI, USA) and sequenced using Big Dye Terminator chemistry by the Australian Genome Research Facility Perth, Western Australia. Sequence identities were confirmed by a Blastn search on the GenBank. Bacterial and archaeal *16 S rRNA*, fungal *18 S rRNA*, LMCO, *cbhI* and *GH48* were isolated from DNA extracted from environmental samples. The standard curves generated in each reaction were linear over seven orders of magnitude (10^8^–10^2^ gene copies) with r^2^ values greater than 0.98. Amplification efficiencies ranged from 90–98% for bacterial *16 S rRNA*, 92–98% for archaeal *16 S rRNA*, 88–98% for fungal *18 S rRNA*, 80–82% for LMCO, 85–98% for *cbhI* and 88–98% for *GH48*.

### Statistical analyses

Data that was normally distributed was not transformed prior to analysis. qPCR data was not normally distributed and was log transformed. Analysis of variance (ANOVA) was used to examine the effects of temperature, nutrients and wetting applications on accumulated CO_2_-C loss, total C, total N, C:N ratio, soil pH (CaCl_2_), NH_4_^+^-N and NO_3_^-^-N concentrations, PMN, MBC, abundance of total bacteria, fungi and archaea and functional genes (LMCO, *cbhI* and *GH48*), and concentration of inorganic P, K and S. Repeated measures ANOVA was used to study the effects of time, wetting applications, nutrient treatments and temperature on daily CO_2_-C production. All statistical analyses were performed in GenStat V16.0 (VSN International, UK).

## Electronic supplementary material

Supplementary tables

## References

[1] Lal R (2008). Sequestration of atmospheric CO2 in global carbon pools. Energy Environ. Sci..

[2] Robertson GP, Paul EA, Harwood RR (2000). Greenhouse gases in intensive agriculture: Contributions of individual gases to the radiative forcing of the atmosphere. Science.

[3] Sponseller RA (2007). Precipitation pulses and soil CO2 flux in a Sonoran Desert ecosystem. Glob. Chang. Biol..

[4] Griffiths E, Birch HF (1961). Microbiological changes in freshly moistened soil. Nature.

[5] Orchard VA, Cook FJ (1983). Relationship between soil respiration and soil-moisture. Soil Biol. Biochem..

[6] Scheu S, Parkinson D (1994). Effects of earthworms on nutrient dynamics, carbon turnover and microorganisms in soils from cool temperate forests of the Canadian Rocky Mountains - laboratory studies. Appl. Soil Ecol..

[7] Adu JK, Oades JM (1978). Physical factors influencing decomposition of organic materials in soil aggregates. Soil Biol. Biochem..

[8] Fierer N, Schimel JP (2002). Effects of drying-rewetting frequency on soil carbon and nitrogen transformations. Soil Biol. Biochem..

[9] Kieft TL, Soroker E, Firestone MK (1987). Microbial biomass response to a rapid increase in water potential when dry soil is wetted. Soil Biol. Biochem..

[10] Rodrigo A, Recous S, Neel C, Mary B (1997). Modelling temperature and moisture effects on C-N transformations in soils: comparison of nine models. Ecol. Modell..

[11] Schwinning S, Sala OE (2004). Hierarchy of responses to resource pulses in and and semi-arid ecosystems. Oecologia.

[12] Creamer CA (2016). Is the fate of glucose-derived carbon more strongly driven by nutrient availability, soil texture, or microbial biomass size?. Soil Biol. Biochem..

[13] Knorr M, Frey SD, Curtis PS (2005). Nitrogen additions and litter decomposition: A meta-analysis. Ecology.

[14] Berg B, Egbert M (1997). Effect of N deposition on decomposition of plant litter and soil organic matter in forest systems. Environ. Rev..

[15] Treseder KK (2008). Nitrogen additions and microbial biomass: a meta-analysis of ecosystem studies. Ecol. Lett..

[16] Janssens IA (2010). Reduction of forest soil respiration in response to nitrogen deposition. Nat. Geosci..

[17] Ramirez KS, Craine JM, Fierer N (2012). Consistent effects of nitrogen amendments on soil microbial communities and processes across biomes. Glob. Chang. Biol..

[18] Craine JM, Morrow C, Fierer N (2007). Microbial nitrogen limitation increases decomposition. Ecology.

[19] Kirkby CA (2013). Carbon-nutrient stoichiometry to increase soil carbon sequestration. Soil Biol. Biochem..

[20] Hatton, T. State of the Environment Report 2011 (Commonwealth of Australia, 2011). Available at: http://www.environment.gov.au/science/soe/2011-report/2-drivers/key-findings. (Accessed: 18th March 2016) (2011).

[21] CSIRO. Climate change in Australia. Available at: http://www.climatechangeinaustralia.gov.au/en/climate-projections/climate-analogues/analogues-explorer/. (Accessed: 18th March 2016) (2015).

[22] Nicholls N (2010). Local and remote causes of the southern Australian autumn-winter rainfall decline, 1958–2007. Clim. Dyn..

[23] Oorts K, Merckx R, Grehan E, Labreuche J, Nicolardot B (2007). Determinants of annual fluxes of CO2 and N2O in long-term no-tillage and conventional tillage systems in northern France. Soil Tillage Res..

[24] Pereira JS (2007). Net ecosystem carbon exchange in three contrasting Mediterranean ecosystems - the effect of drought. Biogeosciences.

[25] Schimel JP, Weintraub MN (2003). The implications of exoenzyme activity on microbial carbon and nitrogen limitation in soil: A theoretical model. Soil Biol. Biochem..

[26] Birch HF (1958). The effect of soil drying on humus decomposition and nitrogen availability. Plant Soil.

[27] Franzluebbers AJ, Haney RL, Honeycutt CW, Schomberg HH, Hons FM (2000). Flush of carbon dioxide following rewetting of dried soil relates to active organic pools. Soil Sci. Soc. Am. J..

[28] Xiang S, Doyle A, Holden PA, Schimel JP (2008). Soil Biology & Biochemistry Drying and rewetting effects on C and N mineralization and microbial activity in surface and subsurface California grassland soils..

[29] Collins HP, Cavigelli MA (2003). Soil microbial community characteristics along an elevation gradient in the Laguna Mountains of Southern California. Soil Biol. Biochem..

[30] Bell C, McIntyre N, Cox S, Tissue D, Zak J (2008). Soil microbial responses to temporal variations of moisture and temperature in a Chihuahuan Desert Grassland. Microb. Ecol..

[31] Paul KI (2003). Defining the relation between soil water content and net nitrogen mineralization. Eur. J. Soil Sci..

[32] Sinclair R (2005). Long-term changes in vegetation, gradual and episodic, on the TGB Osborn Vegetation Reserve, Koonamore, South Australia (1926–2002). Aust. J. Bot..

[33] Parker LW, Freckman DW, Steinberger Y, Driggers L, Whitford WG (1984). Effects of simulated rainfall and litter quantities on desert soil biota: soil respiration, microflora, and protozoa. Pedobiologia (Jena)..

[34] Barnard RL, Osborne CA, Firestone MK (2013). Responses of soil bacterial and fungal communities to extreme desiccation and rewetting. ISME J..

[35] Wolters V (2000). Effects of global changes on above and belowground biodiversity in terrestrial ecosystems: implications for ecosystem functioning. Bioscience.

[36] Prosser JI (2007). The role of ecological theory in microbial ecology. Nat. Rev. Microbiol..

[37] Rui Y (2016). Microbial respiration, but not biomass, responded linearly to increasing light fraction organic matter input: Consequences for carbon sequestration. Sci. Rep..

[38] Grierson PF, Comerford NB, Jokela EJ (1999). Phosphorus mineralization and microbial biomass in a Florida Spodosol: effects of water potential, temperature and fertilizer application. Biol. Fertil. Soils.

[39] Wood JM (2001). Osmosensing and osmoregulatory compatible solute accumulation by bacteria. Comp. Biochem. Physiol. Part A Mol. Integr. Physiol..

[40] Gallo M, Amonette R, Lauber C, Sinsabaugh RL, Zak DR (2004). Microbial community structure and oxidative enzyme activity in nitrogen-amended north temperate forest soils. Microb. Ecol..

[41] Keeler BL, Hobbie SE, Kellogg LE (2009). Effects of long-term nitrogen addition on microbial enzyme activity in eight forested and grassland sites: implications for litter and soil organic matter decomposition. Ecosystems.

[42] Fontaine S, Mariotti A, Abbadie L (2003). The priming effect of organic matter: a question of microbial competition?. Soil Biol. Biochem..

[43] Ramirez KS, Lauber CL, Knight R, Bradford MA, Fierer N (2010). Consistent effects of nitrogen fertilization on soil bacterial communities in contrasting systems. Ecology.

[44] Fierer N (2012). Comparative metagenomic, phylogenetic and physiological analyses of soil microbial communities across nitrogen gradients. Isme J..

[45] IUSS Working Group WRB. *World reference base for soil resources* 2*006*. *World Soil Resources Reports No. 103* **43** (2006).

[46] Wardle DA, Ghani A (1995). A critique of the microbial metabolic quotient (qCO2) as a bioindicator of disturbance and ecosystem development. Soil Biol. Biochem..

[47] Keeney DR, Bremner JM (1966). Comparison and evaluation of laboratory methods of obtaining an index of soil nitrogen availability. Agron. J..

[48] Colwell JD (1963). The estimation of the phosphorus-fertilizer requirements of wheat in southern New-South-Wales by soil analysis. Anim. Prod. Sci..

