# Supplementary material for: Response of microbial biomass and CO2-C loss to wetting patterns are temperature dependent in a semi-arid soil
Source: Sci Rep. 2017 Oct 12;7:13032. doi: 10.1038/s41598-017-13094-9 (PMC5638940; doi:10.1038/s41598-017-13094-9)
Supplement: Supplementary file 1 — Supplementary tables [file 41598_2017_13094_MOESM1_ESM.pdf]

Supplementary Information

Title: Response of microbial biomass and CO_2-C loss to wetting patterns are temperature dependent in a semi-arid soil

Authors: Yichao Rui[1], Deirdre B. Gleeson[1], Daniel V. Murphy[1], Frances C. Hoyle[1, 2, *]

[1] SoilsWest, UWA School of Agriculture and Environment, Faculty of Science, The University of Western Australia, Crawley, WA 6009, Australia. [2] Department of Agriculture and Food Western Australia, South Perth, WA 6151, Australia. [*]Correspondence and requests for materials should be addressed to Frances Hoyle (email: frances.hoyle@uwa.edu.au).

Supplementary Table S1 ANOVA results (*P* value) of the effects of temperature (T), wetting applications and nutrient and their interactions on microbial biomass carbon (MBC), ammonium (NH_4^+-N) and nitrate (NO_3^--N) concentration, potentially mineralisable nitrogen (PMN), inorganic phosphorus (P), potassium (K) and sulphur (S) concentration, pH, total C and nitrogen (N), C:N ratio, and abundance of bacteria, fungi and archaea.

Factors	Cumulative CO_2-C evolution (mg CO_2-C kg^{-1} soil)	MBC (mg C kg^{-1})	NH_4^+-N (mg N kg^{-1})	NO_3^--N (mg N kg^{-1})	PMN (mg N kg^{-1})	Inorganic P (mg P kg^{-1})	Inorganic K (mg K kg^{-1})	Inorganic S (mg S kg^{-1})	Total C (%)	Total N (%)	C:N ratio	Bacteria (copy numbers g^{-1})	Fungi (copy numbers g^{-1})	Archaea (copy numbers g^{-1})
T	<0.001	<0.001	0.998	<0.001	<0.001	<0.001	0.698	<0.001	0.607	0.185	0.011	0.751	0.001	<0.001
Wetting	<0.001	0.047	0.393	0.164	<0.001	0.003	<0.001	0.095	0.015	0.077	0.842	0.478	0.087	<.001
Nutrient	0.011	0.018	0.695	<0.001	0.002	<0.001	0.127	0.482	0.076	<0.001	0.002	0.585	0.087	0.137
T×Wetting	<0.001	<0.001	0.209	0.718	<0.001	0.174	0.100	0.895	0.852	0.506	0.090	0.247	0.214	<0.001
T×Nutrient	<0.001	0.112	0.013	0.477	0.119	0.050	0.140	0.087	0.240	0.097	0.151	0.862	0.999	0.044
Wettings×Nutrient	0.040	0.086	0.250	0.301	0.002	0.139	0.201	0.031	0.864	0.658	0.259	0.329	0.066	0.298
T×Wetting×Nutrient	0.343	0.544	0.534	0.836	0.047	0.099	0.104	0.045	0.417	0.841	0.408	0.560	0.282	0.021

Supplementary Table S2 Primers used in determining gene abundance by qPCR.

Gene	Primer	Sequences (5' – 3')	Fragment Length (bp)	Primer Reference	Cycling condition
Bacterial *16S rRNA*	Eub 338<br>Eub 518	ACTCCTACGGGAGGCAGCAG<br>ATTACCGCGGCTGCTGG	180	Fierer et al. (2005)	As conducted in Fierer et al.,(2005)
Archaeal *16S rRNA*	Arch 915F<br>Arch 519R	CAGCMGCCGCGGTAA<br>GTGCTCCCCCGCCAATTCCT	421	Biddle et al. (2006)	94 ˚C for 10 min then 40 cycles 95 ˚C for 1 min, 56 ˚C for 1 min, 72 ˚C for 1 min, and 78 ˚C for 1 min followed by a melt curve fluorescence data was collected at the 78 ˚ stage.
Fungal *18S rRNA*	FF390<br>FR1GC	CGATAACGAACGAGACCT<br>A4CCATTCAATCGGTA4T	372	Hoshino YT and Morimoto S (2008)	94 ˚C for 10 min then 40 cycles 95 ˚C for 1 min, 50 ˚C for 1 min, 72 ˚C for 1 min, and 78 ˚C for 1 min followed by a melt curve fluorescence data was collected at the 78 ˚ stage.
LMCO	CU1AF<br>CU2R	ACMWCBGTYCAYTGGCAYGG<br>GRCTGTGGTACCAGAANGTNCC	142-155	Kellner et al. (2007)	95 ˚C for 3 min then 40 cycles of: 95˚C for 5 sec, 58 ˚C for 30 sec, followed by a melt curve from 65 ˚C to 95 ˚C.
cbhI	fungcbhIF<br>fungcbhIR	ACCAAyTGCTAyACngGnAA<br>(GC[C,T] TCC CAI AT[A,G] TCC ATC	520-620	Edwards et al. (2008)	95 ˚C for 3 min then 40 cycles of: 95˚C for 5 sec, 60 ˚C for 30 sec, followed by a melt curve from 65 ˚C to 95 ˚C
GH48	GH48_F8<br>GH48_R5	GCCADGHTBGGCGACTACCT<br>CGCCCCABGMSWWGTACCA	150	De Menezes et al. (2015)	95 ˚C for 3 min then 40 cycles of: 95˚C for 5 sec, 58 ˚C for 30 sec, followed by a melt curve from 65 ˚C to 95 ˚C.
